# A Survey of Oncologists’ Perceptions and Opinions Regarding the Use of Granulocyte Colony-Stimulating Factors

**DOI:** 10.1007/s13187-019-01638-8

**Published:** 2019-10-26

**Authors:** Alicia Hawkins, Alysa Murphy, Michelle McNamara, Prasad L. Gawade, Rajesh Belani, Michael A. Kelsh

**Affiliations:** 1 Adelphi Research, Doylestown, PA USA; 2 Amgen Inc., Thousand Oaks, CA USA

**Keywords:** Granulocyte colony-stimulating factors, G-CSF, Oncology Care Model, Febrile neutropenia, Physician survey

## Abstract

The purpose of the study is to describe oncologists’ perceptions and opinions about patient eligibility, guidelines, and barriers for use of granulocyte colony-stimulating factor (G-CSF), overall and stratified by their affiliation with the Oncology Care Model (OCM). In May 2018, we invited and recruited practicing US oncologists from a national database for an online survey. Level of agreement was identified using a seven-point scale, ranging from strongly disagree to strongly agree. Of 200 participating oncologists, 70 were OCM-affiliated. Overall, 65% of oncologists agreed or strongly agreed that all patients at high risk of febrile neutropenia (FN) should receive prophylactic G-CSF, and half agreed or strongly agreed that benefits of G-CSF outweigh the potential adverse effects. The most common barriers to G-CSF use for patients at high risk of FN included patient refusal (37.1% of OCM-affiliated oncologists vs. 21.5% of non-OCM-affiliated oncologists), not on protocol/not supported by guidelines (32.9% vs. 23.1%), lack of reimbursement to practice (30.0% vs. 15.4%), and concerns about insurance coverage (22.9% vs. 26.9%). More OCM-affiliated oncologists reported that their practices offer and strongly encourage adherence to a specific protocol for G-CSF use (49.2%) versus non-OCM oncologists (31.3%). Despite recommendations from national guidelines and strong evidence from randomized, controlled clinical trials, only two thirds of oncologists agree or strongly agree that all patients at high risk of FN should receive primary G-CSF prophylaxis. Decisions about G-CSF prophylaxis may be affected by factors other than risk of FN, such as patient choice, practice protocols/guidelines, lack of reimbursement, and insurance coverage.

**Electronic supplementary material:**

The online version of this article (10.1007/s13187-019-01638-8) contains supplementary material, which is available to authorized users.

## Introduction

Febrile neutropenia (FN) following myelosuppressive chemotherapy is associated with significant morbidity, mortality, dose delays, and dose reductions [1–3]. The risk of FN varies by type of chemotherapy regimen and patient-level risk factors, including older age, poor performance status, low baseline blood cell counts, bone marrow involvement, and prior chemotherapy or radiation [4, 5]. Given the proven effectiveness of granulocyte colony-stimulating factors (G-CSFs) in preventing FN [6–9], the National Comprehensive Cancer Network (NCCN) [10], the Infectious Diseases Society of America (IDSA) [11], the American Society of Clinical Oncology (ASCO) [12], and the American Society of Hematology [13] all recommend G-CSF prophylaxis in patients receiving a chemotherapy regimen associated with a high risk of FN (≥ 20%). For patients receiving regimens associated with intermediate risk of FN (10–20%), the NCCN guidelines recommend considering G-CSF use if at least one patient-level risk factor is present [10].

In the USA, the real-world use of G-CSF among patients at high risk of FN does not reflect the recommendations of national guidelines. G-CSF prophylaxis is observed in 17 to 74% of patients receiving chemotherapy regimens associated with high risk of FN, suggesting underutilization of an important preventive measure [14–16]. There is a paucity of information regarding the decision-making process employed by oncologists in determining the use of G-CSF prophylaxis for appropriate patients. Neither is there evidence about the barriers that oncologists face when deciding to use G-CSF prophylaxis or their understanding of how patient-level risk factors modify FN risk.

The timing of prophylactic pegfilgrastim, the most commonly used G-CSF, is also an issue. National guidelines recommend that pegfilgrastim should be administered 1 to 4 days or 1 to 3 days after the last dose of chemotherapy and that pegfilgrastim administration on the same day as the last dose of chemotherapy is sometimes justified [10, 12]. However, same-day administration is associated with an increased risk of FN compared with optimal dosing on days 1 to 3 following chemotherapy [17]. Thus, most patients must return to the clinic to receive G-CSFs for optimal dosing. An on-body injector (OBI), approved in 2015 [18], can be applied on the same day as chemotherapy and automatically administers pegfilgrastim ~27 h later, allowing patients to avoid a return visit. However, the perceptions and opinions of oncologists related to OBI use have not been studied since 2016, when a survey was conducted among early adopters [19].

Data are also lacking regarding the influence of oncologists’ practice setting. The use of G-CSF may differ in academic centers versus community clinics or in practices participating versus not participating in the Oncology Care Model (OCM). The OCM is a Medicare performance-based payment model that aims to provide higher-quality, more highly coordinated, more patient-centered oncology care at the same or lower cost [20, 21].

The objective of this survey was to describe the perceptions and opinions of US-based oncologists about G-CSF use in patients receiving myelosuppressive chemotherapy. Specifically, we sought to explore oncologists’ perspectives about G-CSF prophylaxis for FN; barriers to G-CSF use across various treatment settings; patient-level risk factors for FN; and differences in G-CSF use, specifically pegfilgrastim, stratified by affiliation with the OCM.

## Methods

### Physician Recruitment

This was a national cross-sectional survey of US oncologists conducted by an independent third party, Adelphi Research. Prior to initiation of the study, the protocol was approved by a centralized, US-based independent review board, Quorum Review (QR#33224/1). After approval, oncologists who currently prescribe G-CSFs were invited and recruited from a national database of 13,800 oncologists hosted by M3 Global Research. Oncologists from Vermont and Maine were excluded because of state prohibitions on compensation for surveys, and oncologists from Massachusetts were excluded due to state requirements for double blinding.

The study targeted recruitment of approximately 200 US oncologists. Targets for specific subgroups of interest included approximately 75 oncologists affiliated with an academic cancer center and approximately 50 oncologists affiliated with the OCM. Oncologists who consented completed a screening section prior to the actual survey so that their eligibility could be assessed according to the following criteria: (1) licensed and board-certified in the USA in oncology and/or hematology for at least 3 years, (2) treated at least 40 unique oncology patients with chemotherapy in the past 6 months, (3) treated at least six oncology patients with a G-CSF in the past 6 months, (4) self or family member not affiliated with a marketing, pharmaceutical, or biotechnology company, and (5) proportion of professional time devoted to patient care ≥ 40% if affiliated with an academic center or ≥ 60% if affiliated with a community center.

### Survey Development

The survey questionnaire was developed using standard survey methodology [22]. Before online implementation, a draft survey questionnaire was reviewed in two pilot tests. The first pilot involved a 60-min face-to-face interview with five oncologists who completed the questionnaire via pen and paper and provided feedback to improve the content and clarity of the questionnaire. During the second pilot, 60-min interviews were conducted with four oncologists via telephone using an online version of the questionnaire, where oncologists provided feedback to improve content and clarity as well as the flow and ease of use of the online questionnaire. Pertinent changes were made to the online questionnaire following each interview based on the feedback.

### Data Collection

The finalized version of the questionnaire consisted of 58 questions: 12 screening questions, 4 demographic questions, 6 questions on G-CSF use overall and by type, 5 questions about predictors of G-CSF use, 23 questions about types of G-CSF selected, and 8 questions about future G-CSF prescribing. The survey collected information on primary affiliation (OCM vs. non-OCM, academic vs. community center), patient-level risk factors, criteria considered to determine a patient’s FN risk level, drivers of and barriers to G-CSF use, and perceptions about G-CSFs. The survey took 30 min to complete (see Online Resource 1 for survey questionnaire).

All survey questions were quantitative, and most were structured to provide mutually exclusive responses. However, some questions had an option to “select all that apply” (e.g., oncologists could choose multiple answers for guidelines they primarily follow regarding G-CSF use [Q.B1a] or factors they consider to be barriers to G-CSF use [Q.B3]). The level of agreement was identified in the questionnaire using a seven-point scale, where responses from 1 to 7 indicated strongly disagree, disagree, somewhat disagree, neither agree nor disagree, somewhat agree, agree, and strongly agree, respectively. To minimize missing data, the electronic questionnaire was designed in such a way that a question had to be completed before the subsequent question was made available to the participant.

### Statistical Analysis

Continuous variables were summarized as mean, standard deviation, median, and interquartile range, and categorical variables were summarized as number and percentage with binomial 95% confidence interval (CI). Outliers were identified as values beyond 3 standard deviations and excluded from the analyses. The seven-point scale for level of agreement was categorized into three groups: strongly disagree or disagree; somewhat disagree, neither agree nor disagree, or somewhat agree; and agree or strongly agree. To understand the differences between groups, survey data was further stratified by OCM and academic center affiliation. Analyses were conducted using Q Research Software (https://www.qresearchsoftware.com).

## Results

### Demographics

A total of 337 oncologists volunteered to participate in the survey before the target of 200 oncologists was reached. Of the 337 oncologists, 89 were found to be ineligible according to the screening questions and 48 did not complete the survey. A total of 200 oncologists completed the online survey from May 2018 to June 2018, of whom 114 (57.0%) were hematologist-oncologists and 86 (43.0%) were medical oncologists.

Overall, oncologists reported practicing for an average of 13 years, and each participating oncologist provided G-CSF support for an average of approximately 80 patients in the past 6 months. The most common practice settings reported by oncologists were group practices (36.0%) and major academic centers (25.0%). The majority of the 200 oncologists were primarily affiliated with non-OCM practices (*n* = 130; 65.0%) and community clinics (*n* = 125; 62.5%). Detailed characteristics of the participating oncologists stratified by institutional affiliation are presented in Table 1.

**Table 1:** Characteristics of US oncologists participating in the online survey stratified by institutional affiliation, May–June 2018

Characteristic	OCM-affiliated (*n* = 70)			Non-OCM-affilliated (*n* = 130)			Total (*N* = 200)
	**Total** (*n* = 70)	**Academic** (*n* = 28)	**Community** (*n* = 42)	**Total** (*n* = 130)	**Academic** (*n* = 47)	**Community** (*n* = 83)	
Hematology oncology	41 (58.6%)	15 (53.6%)	26 (61.9%)	73 (56.2%)	24 (51.1%)	49 (59.0%)	114 (57.0%)
Medical oncology	29 (41.4%)	13 (46.4%)	16 (38.1%)	57 (43.8%)	23 (48.9%)	34 (41.0%)	86 (43.0%)
Number of patients treated with a G-CSF in past 6 months; median (IQR)a	83 (79)	60 (60)	90 (127)	80 (75)	80 (85)	78 (75)	80 (75)
Years in practice; mean (SD)	12.5 (6.8)	11.6 (6.7)	13.1 (6.9)	13.2 (7.6)	11.7 (7.7)	14.1 (7.5)	13.0 (7.3)
Practice setting							
Group practice	27 (38.6%)	0 (0.0%)	27 (64.3%)	45 (34.6%)	0 (0.0%)	45 (54.2%)	72 (36.0%)
Major academic left	15 (21.4%)	15 (53.6%)	0 (0.0%)	35 (26.9%)	35 (74.5%)	0 (0.0%)	50 (25.0%)
Outpatient clinic	9 (12.9%)	0 (0.0%)	9 (21.4%)	20 (15.4%)	0 (0.0%)	20 (24.1%)	29 (14.5%)
Teaching hospital affiliated with a university medical school	13 (18.6%)	13 (46.4%)	0 (0.0%)	12 (9.2%)	12 (25.5%)	0 (0.0%)	25 (12.5%)
Community hospital (non-teaching)	6 (8.6%)	0 (0.0%)	6 (14.3%)	15 (11.5%)	0 (0.0%)	15 (18.1%)	21 (10.5%)
Solo practice	0 (0.0%)	0 (0.0%)	0 (0.0%)	3 (2.3%)	0 (0.0%)	3 (3.6%)	3 (1.5%)
Geographic region of practice							
Northeast	18 (25.7%)	11 (39.3%)	7 (16.7%)	30 (23.1%)	14 (29.8%)	16 (19.3%)	48 (24.0%)
Midwest	15 (21.4%)	6 (21.4%)	9 (21.4%)	31 (23.8%)	9 (19.1%)	22 (26.5%)	46 (23.0%)
South	26 (37.1%)	9 (32.1%)	17 (40.5%)	42 (32.3%)	14 (29.8%)	28 (33.7%)	68 (34.0%)
West	20 (28.6%)	5 (17.9%)	15 (35.7%)	33 (25.4%)	12 (25.5%)	21 (25.3%)	53 (26.5%)

Data presented as *n* (%) of oncologists unless otherwise specified

*G-CSF* granulocyte colony-stimulating factor, *IQR* interquartile range, *OCM* Oncology Care Model, *SD* standard deviation

^a^Results for this question are based on *n* = 193 oncologists (*n* = 64 OCM, *n* = 129 non-OCM) after excluding seven outliers

### Adherence to Guidelines

In response to the question regarding what guidelines they follow for G-CSF use (Q.B1a), most oncologists reported awareness of the NCCN guidelines (70.5%), followed by ASCO guidelines (38%), institution-specific or practice-specific guidelines (17%), American Society of Hematology guidelines (12%), no guidelines (11%), Centers for Disease Control and Prevention guidelines (6%), and other (2%).

Almost two thirds of oncologists agreed or strongly agreed with the statement that patients at high risk of FN should receive primary G-CSF prophylaxis. According to practice setting, the figures were 57.3% for academic center, 69.6% for community practice, 60.0% for OCM-affiliated, and 67.7% for non-OCM-affiliated. More OCM oncologists reported that their practices strongly encourage adherence to a specific treatment protocol for G-CSF use (49.2% [95% CI 37.3%, 61.2%]) compared with non-OCM oncologists (31.3% [95% CI 23.5%, 40.3%]). Additionally, oncologists affiliated with practices where specific treatment protocols are strongly encouraged were more likely to say they use G-CSF for primary prophylaxis for patients in the adjuvant chemotherapy setting (53.6% of patients [95% CI 46.6%, 60.7%]) compared with oncologists affiliated with practices that somewhat encourage specific treatment protocols (41.7% of patients [95% CI 35.4%, 48.0%]).

Participants were asked to select the recommended timing for administering pegfilgrastim prefilled syringe (PFS) from four predefined timeframes. The majority of the oncologists in both the OCM (72.5%) and non-OCM (75.0%) subgroups indicated that pegfilgrastim PFS should be administered between 24 and 48 h following completion of chemotherapy administration. Almost a quarter of oncologists in both the OCM (23.5%) and non-OCM (22.8%) subgroups said that the recommended timing of pegfilgrastim PFS is within 24 h of the last day of chemotherapy administration (Table 2).

**Table 2:** Timing of pegfilgrastim PFS use as reported by the 143 US oncologists who reported that they had prescribed pegfilgrastim PFS in the past 6 months, stratified by institutional affiliation

Timing of PFS administration	OCM-affiliated (*n* = 51)			Non-OCM-affiliated (*n* = 92)			Total (*N* = 143)
	**Total** (*n* = 51)	**Academic** (*n* = 17)	**Community** (*n* = 34)	**Total** (*n* = 92)	**Academic** (*n* = 29)	Community **(*n*= 63)**	
What is the current recommended timing for administering pegfilgrastim PFS?, *n* (%)							
Within 24 h of the last day of chemotherapy administration	12 (23.5%)	2 (11.8%)	10 (29.4%)	21 (22.8%)	4 (13.8%)	17 (27.0%)	33 (23.1%)
Between 24 and 48 h after last the day of chemotherapy administration	37 (72.5%)	13 (76.5%)	24 (70.6%)	69 (75.0%)	24 (82.8%)	45 (71.4%)	106 (74.1%)
2–4 days after the last day of chemotherapy administration	2 (3.9%)	2 (11.8%)	0 (0.0%)	2 (2.2%)	1 (3.4%)	1 (1.6%)	4 (2.8%)
5 or more days after last day of chemotherapy administration	0 (0.0%)	0 (0.0%)	0 (0.0%)	0 (0.0%)	0 (0.0%)	0 (0.0%)	0 (0.0%)

*PFS* prefilled syringe, *OCM* Oncology Care Model

### Importance of Factors in Determining High Risk of Developing FN

**Fig. 1:**
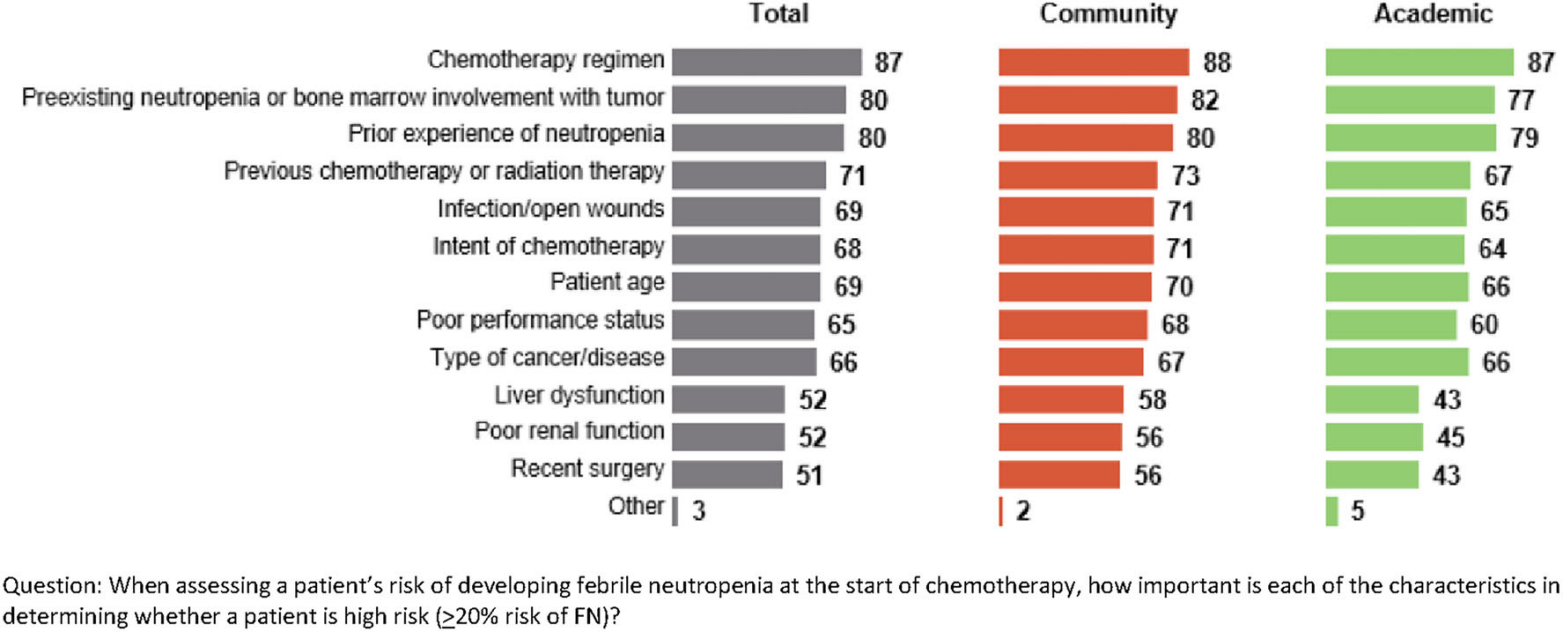
Mean ratings of factors used to determine high risk of febrile neutropenia reported by 200 US oncologists, stratified by affiliation with community or academic practice

Oncologists were asked to consider the importance of 12 predefined factors in determining whether a patient is at high risk of developing FN at the start of chemotherapy. The rating of these factors was done on a scale of 0 (least important) to 100 (most important). Based on the mean ratings, the factors considered most important in determining whether a patient is at high risk of developing FN were chemotherapy regimen, preexisting neutropenia or bone marrow involvement with tumor, and prior experience of neutropenia (Fig. 1). Several risk factors for FN were rated higher by oncologists primarily affiliated with community centers than those affiliated with academic centers, including liver dysfunction (57.9 [95% CI 53.6, 62.2] vs. 43.1 [95% CI 37.2, 48.9]), poor renal function (56.5 [95% CI 52.0, 60.9] vs. 44.7 [95% CI 38.7, 50.6]), and recent surgery (55.9 [95% CI 51.5, 60.2] vs. 42.7 [95% CI 36.6, 48.7]). Ratings of factors for developing FN did not differ between OCM and non-OCM oncologists.

### Perceptions and Opinions About G-CSF Use and Barriers to Use

**Fig. 2:**
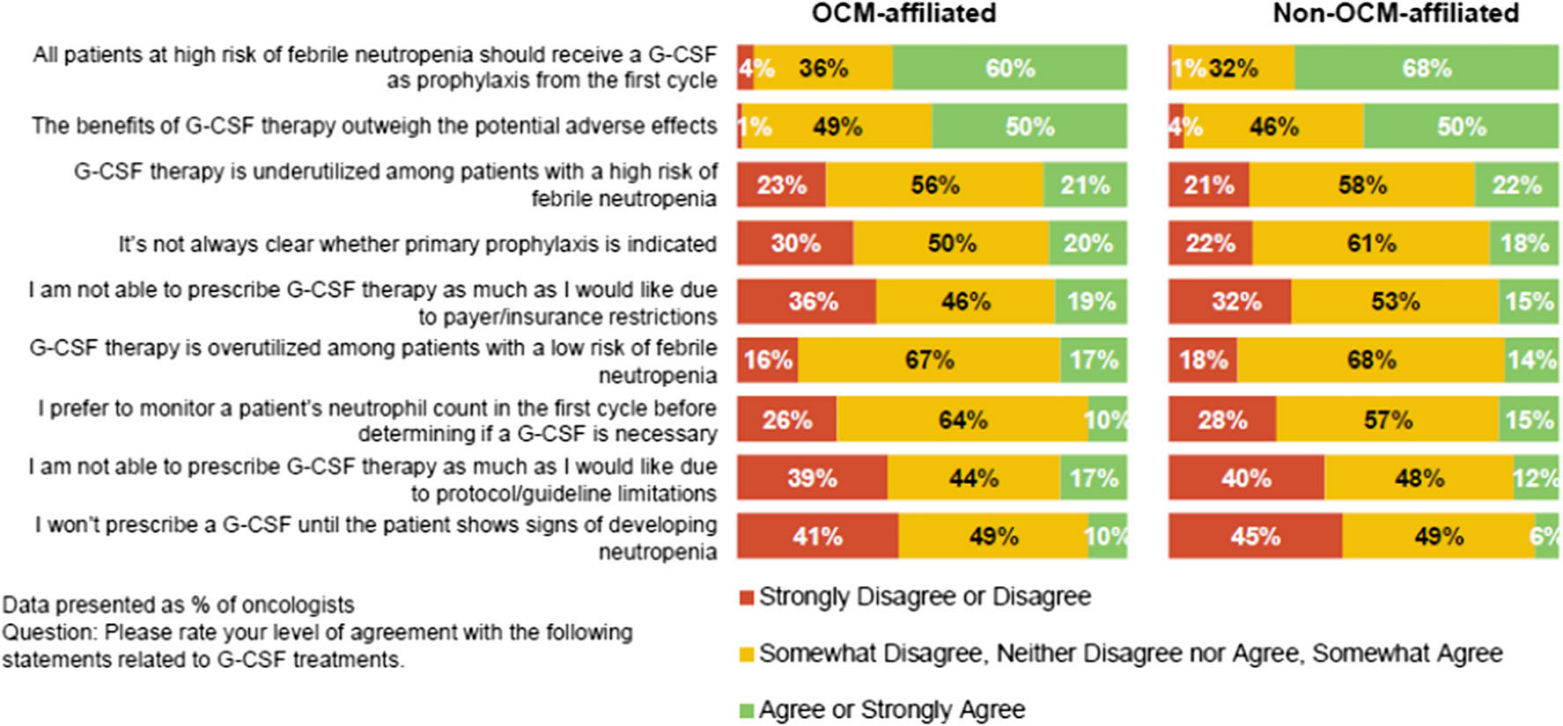
Perceptions and opinions of 200 US oncologists about use of granulocyte colony-stimulating factor (G-CSF), stratified by affiliation with Oncology Care Model (OCM) practice

When asked for their opinions about G-CSF prescribing, 65% of the oncologists either agreed or strongly agreed that all patients at high risk of FN should receive prophylaxis with G-CSF, with the level of agreement being somewhat higher among non-OCM than OCM oncologists (67.7% [95% CI 59.2%, 75.1%] vs. 60% [95% CI 48.3%, 70.7%]). Additionally, half of oncologists (50.0%) agreed or strongly agreed that the benefits of G-CSF therapy outweigh the potential adverse effects, with no difference observed according to OCM affiliation (Fig. 2).

Overall, the most commonly reported barriers to prescribing G-CSF as primary prophylaxis for patients at high risk of FN included patient refusal (27.0%), not being on protocols set by the practice or guidelines (26.5%), and concerns about insurance coverage (25.5%). OCM oncologists perceived barriers to prescribing G-CSF as prophylaxis differently than non-OCM oncologists did, with more OCM oncologists than non-OCM oncologists reporting lack of reimbursement to the practice/organization (30% [95% CI 20.5%, 41.6%] vs. 15.4% [95% CI 10.1%, 22.6%]) and patient refusal (37.1% [95% CI 26.8%, 48.9%] vs. 21.5% [95% CI 15.3%, 29.4%]) as barriers (Table 3).

**Table 3:** Barriers to prescribing prophylactic G-CSF to patients receiving chemotherapy with high risk of FN, stratified by oncologist’s affiliation with OCM practice

Barrier	Total (*N* = 200)		OCM-affiliated (*n* = 70)		Non-OCM-affiliated (*n* = 130)	
	*n*	% (95% CI)	*n*	% (95% CI)	*n*	% (95% CI)
Not on protocol/not supported by guidelines	53	26.5% (20.9%, 33.0%)	23	32.9% (23.0%, 44.5%)	30	23.1% (16.6%, 31.1%)
Cost to patient	52	26.0% (20.4%, 32.5%)	17	24.3% (15.7%, 35.6%)	35	26.9% (20.0%, 35.2%)
Safety/side effect concerns	27	13.5% (9.4%, 19.0%)	9	12.9% (6.7%, 22.9%)	18	13.8% (8.9%, 20.9%)
Concerns about insurance coverage	51	25.5% (19.9%, 32.0%)	16	22.9% (14.5%, 34.0%)	35	26.9% (20.0%, 35.2%)
Lack of reimbursement to practice/organization	41	20.5% (15.5%, 26.7%)	21	30.0% (20.5%, 41.6%)	20	15.4% (10.1%, 22.6%)
Patient inconvenience	23	11.5% (7.7%, 17.6%)	11	15.7% (8.8%, 26.2%)	12	9.2% (5.2%, 15.6%)
Patient refusal	54	27.0% (21.3%, 33.6%)	26	37.1% (26.8%, 48.9%)	28	21.5% (15.3%, 29.4%)
I prefer to wait and see	17	8.5% (5.3%, 13.3%)	8	11.4% (5.7%, 21.2%)	9	6.9% (3.5%, 12.8%)
Other	1	0.5% (− 0.2%, 3.1%)	0	0% (− 1.0%, 6.2%)	1	0.8% (− 0.3%, 4.7%)
There are no barriers	66	33.0% (26.9%, 39.8%)	18	25.7% (16.9%, 37.1%)	48	36.9% (29.1%, 45.5%)

*FN* febrile neutropenia, *G-CSF* granulocyte colony-stimulating factor, *OCM* Oncology Care Model

### Changes in Use of Pegfilgrastim

When asked to rate on a five-point scale how their use of pegfilgrastim OBI has changed over the past 2 years (decreased significantly, decreased somewhat, same, increased somewhat, increased significantly), 67.0% of oncologists reported that the use was increased somewhat or significantly. Subsequent questions, which allowed oncologists to select multiple reasons for increased use of OBI, showed that oncologists most commonly attributed the increase to convenient dosing (66.7%) and patients’ need for an active lifestyle (45.5%). Non-OCM oncologists reported convenient dosing as an influential factor more frequently than OCM oncologists (72.6% [95% CI 62.2%, 81.1%] vs. 56.3% [95% CI 42.3%, 69.3%]).

## Discussion

The majority of the oncologists in this survey reported adhering to at least one national guideline for G-CSF use [10–12], but their responses did not exactly align with these guidelines. National guidelines recommend that patients at high risk of FN should receive primary G-CSF prophylaxis, but only two thirds of participating oncologists agreed or strongly agreed with this statement. Despite the clinical evidence presented in national guidelines, only half of the oncologists agreed or strongly agreed that the benefits of G-CSF outweigh the adverse events. The lack of strong agreement with national guidelines with respect to who is eligible for G-CSF prophylaxis and the risk–benefit ratio of G-CSF aligns with existing literature, which shows disparity between the recommended and real-world use of G-CSF [23].

Furthermore, almost a quarter of the oncologists in this survey said that the recommended timing of pegfilgrastim PFS administration is within 24 h of the last dose of chemotherapy administration. This misinterpretation of recommended timing could be one of the reasons for almost 12% of chemotherapy cycles in the real-world setting being given with same-day pegfilgrastim administration [17].

The reasons for same-day pegfilgrastim administrations are multifactorial and have been explored in a survey of oncologists who prescribed at least one same-day dose of pegfilgrastim in the past 6 months [24]. Previous FN, presence of infection or open wounds, poor performance status, and patient age were reported as the most common clinical considerations for same-day administration. Both NCCN and ASCO guidelines reference the availability of an automated injection device (pegfilgrastim OBI) for patients who cannot return to the clinic for next-day pegfilgrastim administration [10, 12]. In the current study, an increase in use of pegfilgrastim OBI over the last 2 years was reported by two thirds of the oncologists, and they most commonly attributed this increase to convenient dosing and patients’ need for an active lifestyle. These results reaffirm the findings from a 2016 survey of 200 patients and 200 oncologists, which found that avoiding the need to return to the clinic was the most important treatment feature in the opinion of both patients and oncologists [19].

Compared with non-OCM oncologists, OCM oncologists were more likely to have their practices offer or strongly encourage adherence to a specific protocol for G-CSF use. The OCM sites may be more likely to adhere to a specific protocol for G-CSF use because the practice performance is graded not only on outcomes such as all-cause hospitalization and emergency room visits but also on medication use [25]. Another difference between OCM and non-OCM oncologists was that lack of reimbursement to practice and patient refusal were more commonly reported by OCM oncologists as barriers to G-CSF use. However, only 60% of OCM and 68% of non-OCM oncologists felt strongly that all patients at high risk of FN should receive prophylaxis with G-CSF.

Compared with academic oncologists, community oncologists rated hepatic and renal dysfunction and recent surgery as more important determinants of high risk of FN. The perception of risk factors for FN may be different for community oncologists compared with academic oncologists, and this may explain the slightly higher use of G-CSF for primary prophylaxis reported by community oncologists in this study. This finding is similar to previous survey studies of ASCO oncologists, which detected greater use of prophylactic G-CSF by oncologists practicing in a fee-for-service setting [26, 27].

We restricted the study to US oncologists who met the eligibility criteria, volunteered to participate in an online survey, and had used G-CSFs in their practice. Thus, the results may not be generalizable to non-US oncologists, to US oncologists who were ineligible to participate, or to oncologists who do not use G-CSFs yet treat patients who are otherwise eligible for prophylactic FN treatment. The selection of oncologists using physician panel is not truly random, and those volunteering to participate may not be representative of source population of oncologists. The survey collected information on oncologists’ perceptions that were based on recall; therefore, the results may not reflect routine practice. Substantial variations in patterns of G-CSF use have been noted between oncologists belonging to the same community practice [28]; however, we believe that a sample size of 200 oncologists should provide valid average directionality of the practice patterns. The results are meant to be descriptive, and for certain subgroups, the small sample size limits our ability to make comparisons.

## Conclusion

Despite G-CSF approval since 1991 and the availability of evidence-based guidelines, FN prophylaxis in appropriate patients remains uneven. Our survey shows that not all oncologists follow national guidelines. Noncompliance with prophylactic G-CSF use in appropriate patients can increase FN risk, leading to increased morbidity and potentially increased health care costs. Further education of oncologists on the downstream consequences of not adhering to national guidelines could help reduce the underutilization and inappropriate timing of G-CSF, consequently reducing the risk of FN. Pegfilgrastim OBI is now increasingly used for next-day pegfilgrastim injection, reducing the travel burden and likely reducing the risk of noncompliance as a result of the travel burden. Barriers to G-CSF, including concerns about lack of reimbursement and patient refusal, differ by OCM affiliation and need to be explored further and overcome to improve coverage for G-CSF prophylaxis in appropriate patients.

## Electronic supplementary material

ESM 1 (DOCX 230 kb)
