# Supplementary material for: A Survey of Oncologists’ Perceptions and Opinions Regarding the Use of Granulocyte Colony-Stimulating Factors
Source: J Cancer Educ. 2019 Oct 26;35(1):178–86. doi: 10.1007/s13187-019-01638-8 (PMC6971139; doi:10.1007/s13187-019-01638-8)
Supplement: Supplementary file 1 — (DOCX 230 kb) [file 13187_2019_1638_MOESM1_ESM.docx]

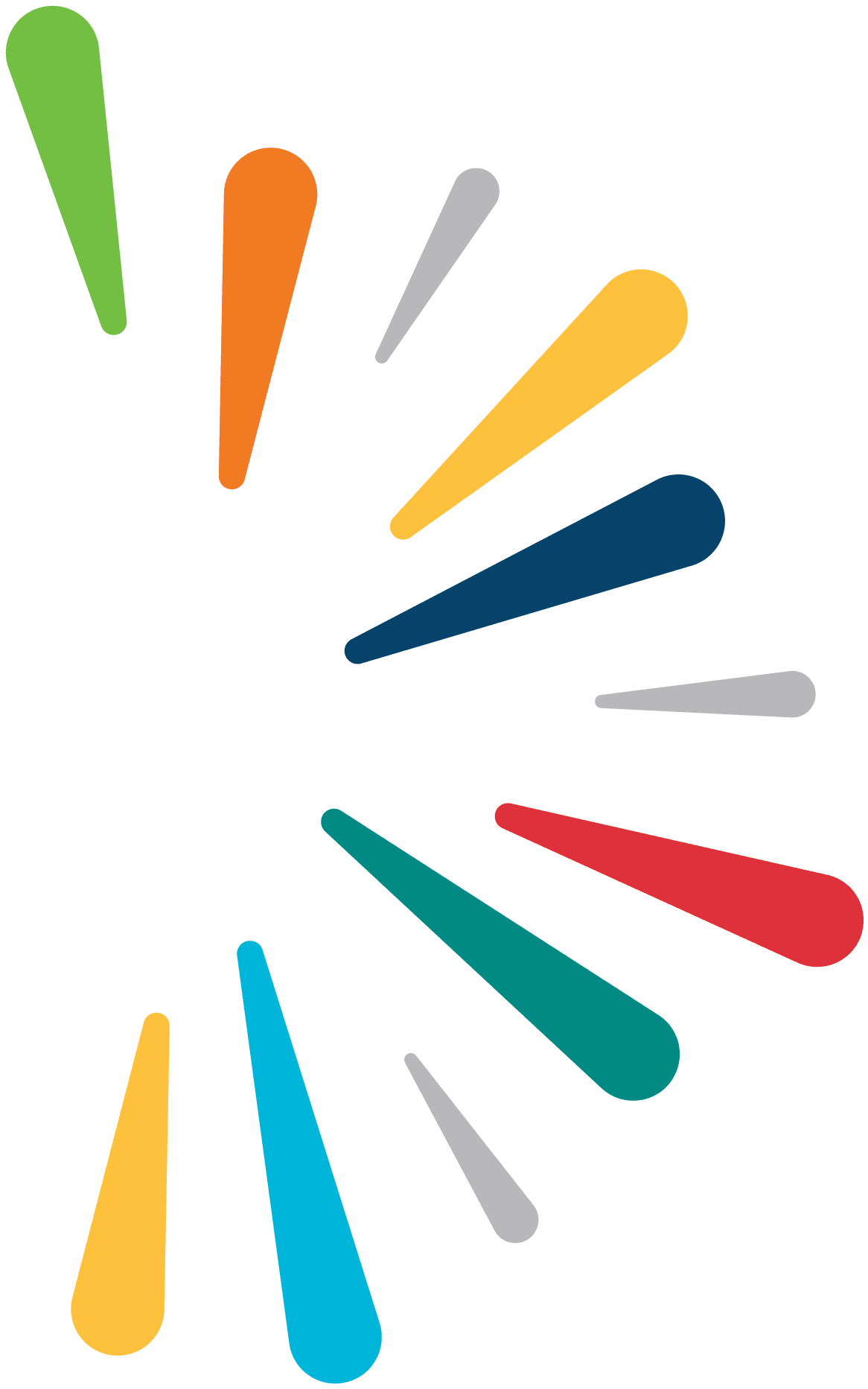


**GCSF Decision Making Survey
Screener and Questionnaire**

DRAFT: May 4, 2018

Project #: AM8636

Planisware: 20180068

# SCREENER

|  |  | ONCs/HEMs |  |
| --- | --- | --- | --- |
| Q  U  O  T  A  S | TOTAL | N=200 |  |
|  | Community | N=125 |  |
|  | Academic | N=75 |  |
|  | Oncology Care Model (OCM) | N=50 |  |

Thank you for your interest in participating in this online study focusing on the treatment of cancer patients. Please review and approve the statement below to participate in this study.

Adelphi Research wishes to confirm your interest in participating in this research. This Participant Agreement (“Agreement”) sets forth the terms and conditions of your participation.

Research Intent. Any information provided to you is for market research purposes only and is not intended to recommend or promote a therapeutic approach, medical procedure, specific product or class of products, or to be a representation of approved product labeling.  You have no obligation to use, purchase, recommend or arrange for the use of any therapeutic approach, medical procedure, or product, based on your participation in this market research. You represent that the facts and information that you provide in the course of your participation are true and accurate.

Confidentiality. You agree to keep in confidence for a period of three (3) years from the expiration or termination of this Agreement any confidential information disclosed to you during this discussion.  “Confidential Information” includes, but is not limited to, information about the Client (if applicable) or the subject of the research that is either proprietary in nature or is not known to the general public.

Your responses and any other information you provide in the context of your participation in this research will be kept confidential and will only be used as described in this document and will not be disclosed to any third party (other than those described in this document) without your approval.

Payment. As consideration for the time you are taking to participate in this market research and for your performance of the obligations under this Agreement, we shall pay you the amount that was previously communicated in your invitation email.

Disclosure Requirements.

In the event that the Client is required under local law or industry requirements, including, without limitation, the U.S. Sunshine Act and applicable U.S. state transparency laws, Client will publicly disclose information relating to your participation under this Agreement, including, without limitation, your name, address, the nature of the services performed and any and all payments, reimbursements for expenses, or other transfers of value made to you under this Agreement.

Disclaimer:

The brands listed are the registered trademarks of their respective owners. Neulasta®, Neulasta® Onpro® and NEUPOGEN® are registered trademarks of Amgen Inc. GRANIX® is a registered trademark of Teva Pharmaceutical Industries Ltd. ZARXIO® is a registered trademark of Novartis AG.

| 1 | Approve | 2 | Disapprove |
| --- | --- | --- | --- |

We are now being asked to pass on to our client details of adverse events that are raised during the course of market research interviews. Although this is an on-line market research interview and how you respond will, of course, be treated in confidence, should you raise an adverse event and / or product complaint, we will need to report this, even if it has already been reported by you directly to the company or the regulatory authority. The Adverse Event data collected may be sent outside the reporting country for processing.

We will initially forward the Adverse Report to the client’s pharmacovigilance department anonymously. Any requests for further information from pharmacovigilance will come through the recruiting agency.

| 1 | I would like to proceed and give permission for the recruitment agency to contact me to provide more information about an adverse event mentioned by me during the survey. |
| --- | --- |
| 2 | I would like to proceed and DO NOT want the recruitment agency to contact me to provide more information about an adverse event mentioned by me during the survey. |
| 3 | I don’t want to proceed and end the interview here |

We may want to further explore your responses to some of the questions answered. Would you be prepared to be re-contacted to take part in further research?

| 1 | Yes | 2 | No |
| --- | --- | --- | --- |

By proceeding to the next screen, I confirm that I have read, understood and accept the statements presented in the previous screens and I am happy to proceed with the market research survey on this basis.

Select one only.

| 1 | YES I am happy to proceed with the market research survey on this basis |
| --- | --- |
| 2 | NO I am not happy to proceed with the market research survey on this basis and I do not wish to continue. |

S0

Are you or is any member of your family employed by or otherwise affiliated with a marketing, advertising, public relationships, media, medical research, pharmaceutical, or biotech company?

| 1 | Yes |  |
| --- | --- | --- |
| 2 | No |  |

First, please answer a few questions to confirm your eligibility. We are looking for your thoughtful responses—***please take the time to read each question and answer as honestly and accurately as possible.***

S1

Which of the following best describes your primary medical specialty?

| 1 | Medical oncology |  |
| --- | --- | --- |
| 2 | Hematology oncology |  |
| 3 | Radiation oncology |  |
| 4 | Neuro-oncology |  |
| 5 | Gynecological oncology |  |
| 7 | Surgical oncology |  |
| 8 | Other (specify: _______________) |  |

S2

Are you board certified or board-eligible in your specialty?

| 1 | Yes |  |
| --- | --- | --- |
| 2 | No |  |

S3a

In which states do you currently reside? ***Select all that apply***
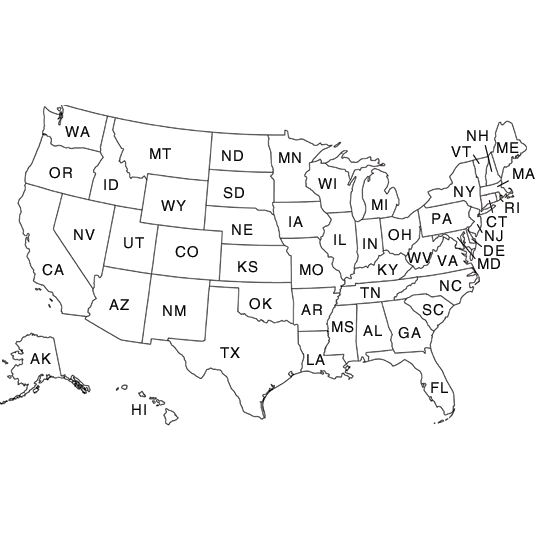


S3b

Which states are you licensed to practice in at this time? ***Select all that apply.***
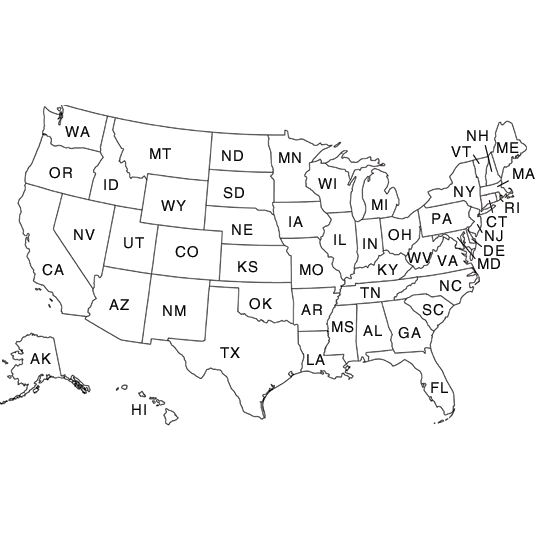


S4.

How many years have you been in practice, post-residency?

_____years

S5.

What percentage of your professional time is spent in each of the following settings, and which one of these describes the practice setting where you spend the majority of your time seeing patients?

*Please enter a response in the first column and select one option in the second column.*

|  |  | % of Professional Time | Primary practice setting |
| --- | --- | --- | --- |
| 1 | Major academic center | ____ | ○ |
| 2 | Teaching hospital affiliated with a university medical school | ____ | ○ |
| 3 | Community hospital (non-teaching) | ____ | ○ |
| 4 | Group practice | ____ | ○ |
| 5 | Solo practice | ____ | ○ |
| 7 | Outpatient clinic | ____ | ○ |
| 6 | Government-owned (e.g., VA, DoD, state, etc.) | ____ | ○ |
| 95 | Other (specify) | ____ | ○ |

S5b.

Which of the following best describes your affiliation with an academic/teaching hospital? *Select one.*

| 1 | I am an employee and I hold a faculty position at an academic center or university hospital |  |
| --- | --- | --- |
| 2 | I have a faculty position at an academic center or university hospital, but am not otherwise employed by the center |  |
| 3 | I am an employee at an academic center or university hospital, but do not hold a faculty position |  |
| 4 | I am not an employee or faculty member at a university hospital or academic center, but I sometimes have teaching or clinical obligations there |  |
| 5 | I have no formal position at an academic center or university hospital |  |
| 95 | Other (specify): |  |

S6.

Is your primary practice center a part of the Oncology Care Model (OCM)?

| 1 | Yes |  |
| --- | --- | --- |
| 2 | No |  |

S7.

Are you personally responsible for the initiation of chemotherapy for oncology patients?

| 1 | Yes |  |
| --- | --- | --- |
| 2 | No |  |

S8.

What percentage of your professional time is spent directly caring for patients (as opposed to teaching or administration)?

_____% of time in direct care

S9.

Approximately how many unique patients in total with any condition did you personally manage across all settings over the **past** **6 months**?

_____ # of patients seen or treated in the past 6 months

S10.

Of the [insert S9] patients seen in the past 6 months, how many unique patients have you seen for neoplasms (solid tumors and hematologic malignancies) as opposed to other conditions?

|  |  | # of patients seen/treated  in past 6 months |
| --- | --- | --- |
| 1 | Unique patients with solid tumors | ____ |
| 2 | Unique patients with hematologic malignancies | ____ |
| 3 | Unique patients with other conditions | ____ |

S11.

Of your [S10_1] **solid tumor and [**S10_2] **hematologic tumor** patients seen or treated in the past 6 months, how many **completed a course of chemotherapy** within the past 6 months?

_____ # of unique solid and hematologic tumor patients seen or treated who completed a course of chemotherapy in the past 6 months

S12.

Of the [insert s11] patients that have completed a course of chemotherapy within the past 6 months, approximately how many received a **granulocyte-colony stimulating factors (G-CSF)** at some point during their chemotherapy?

_____ # of unique patients treated with a G-CSF in the past 6 months

Congratulations, you qualify to participate in our study. This survey has been **time-tested** and takes about 30 minutes to complete. Your responses may be selected for review as part of our on-going quality assurance initiatives. We ask that you give thoughtful answers to all of your questions; inconsistent or illogical responses or completing the survey in significantly less time may result in forfeiture of honorarium.

❑ I have read and understand the statement above. (Must check to continue)

: At this time, you do not qualify to participate in this survey. Thank you very much for your interest.

# Section A: G-CSF Usage

First, we would like to understand your overall approach and usage of granulocyte-colony stimulating factor, which we will refer to as **G-CSF** for the remainder of this survey.

For this section, please think about the [insert s11] patients you have treated with chemotherapy **in the past 6 months.**

A1.

Thinking about all the patients you have treated with chemotherapy in the past 6 months in the adjuvant or advanced/metastatic setting, what proportion were treated in each setting?

|  |  | % of Patients Treated with Chemotherapy in Past 6 Months in Each Setting |
| --- | --- | --- |
| 1 | Adjuvant setting |  |
| 2 | Advanced/Metastatic setting |  |
|  | TOTAL |  |

*Please enter a number between 0 and 100 for each row.* Your responses must add to 100%.

A3.

For your patients treated with chemotherapy in the past 6 months in each setting who received G-CSF therapy, what percentage received G-CSF as each type of use?

Primary Prophylaxis – to prevent neutropenia: G-CSF prescribed in the 1^st^ chemotherapy cycle and all subsequent cycles
Secondary Prophylaxis – to prevent further neutropenia: G-CSF prescribed in all subsequent chemotherapy cycles after the patient experienced neutropenia/low neutrophil count
Reactive Treatment – to treat neutropenia: G-CSF only prescribed in cycles where patient experiences neutropenia

*Please enter a number between 0 and 100 for each row.* Your responses must add to 100% in each column.

|  |  | % of Patients in Adjuvant Setting |  | % of Patients in Advanced/ Metastatic Setting |
| --- | --- | --- | --- | --- |
| 1 | Received G-CSF as **Primary** Prophylaxis |  |  |  |
| 2 | Received G-CSF as **Secondary** Prophylaxis |  |  |  |
| 3 | Received G-CSF as **Reactive** Treatment |  |  |  |
| 4 | Received **No** G-CSF Therapy |  |  |  |
|  | TOTAL |  |  |  |

A4.

Now, again thinking back to all the patients you have treated with chemotherapy in the past 6 months, what percent would you estimate fell into the following febrile neutropenia (FN) risk categories before starting chemotherapy?

*Please enter a number between 0 and 100 for each row.* Your responses must add to 100%

|  |  | % of Patients Treated with Chemotherapy in Past 6 Months at Each Initial Risk Level |
| --- | --- | --- |
| 1 | High Risk (>20% risk for FN) |  |
| 2 | Intermediate Risk (10-20% risk for FN) |  |
| 3 | Low Risk (<10% risk for FN) |  |
|  | TOTAL |  |

A6.

For your patients treated with chemotherapy in the past 6 months at each FN risk level before starting chemotherapy who received G-CSF therapy, what percentage received G-CSF as each type of use?

Primary Prophylaxis – to prevent neutropenia: G-CSF prescribed in the 1^st^ chemotherapy cycle and all subsequent cycles
Secondary Prophylaxis – to prevent further neutropenia: G-CSF prescribed in all subsequent chemotherapy cycles after the patient experienced neutropenia/low neutrophil count
Reactive Treatment – to treat neutropenia: G-CSF only prescribed in cycles where patient experiences neutropenia

*Please enter a number between 0 and 100 for each row.* Your responses must add to 100% in each column.

|  |  | % of High Risk Patients |  | % of Intermediate Risk Patients |  | % of Low Risk Patients |
| --- | --- | --- | --- | --- | --- | --- |
| 1 | Received G-CSF as **Primary** Prophylaxis |  |  |  |  |  |
| 2 | Received G-CSF as **Secondary** Prophylaxis |  |  |  |  |  |
| 3 | Received G-CSF as **Reactive** Treatment |  |  |  |  |  |
| 4 | Received **No** G-CSF Therapy |  |  |  |  |  |
|  | TOTAL |  |  |  |  |  |

A7.

Previously you indicated you have seen or treated **[insert s11]** total patients that have completed a course of chemotherapy within the past 6 months.

Of those **[insert s11]** patients, how many had each of the following primary tumor types?
*Please note your total may be greater than [S11].*

|  |  | # Patients in Past 6 Months |
| --- | --- | --- |
| 1 | Breast Cancer | ____ |
| 2 | Colorectal Cancer | ____ |
| 3 | Non-Small Cell Lung Cancer | ____ |
| 4 | Ovarian Cancer | ____ |
| 5 | Other solid tumors | ____ |
| 6 | Non-Hodgkin’s Lymphoma | ____ |
| 7 | Other hematologic malignancies | ____ |
|  |  |  |

**A9.**

For your patients treated with chemotherapy in the past 6 months, **for each primary tumor type** who received **G-CSF therapy**, what percentage received G-CSF as each **type of use**?

**Primary Prophylaxis – to prevent neutropenia**: G-CSF prescribed in the 1^st^ chemotherapy cycle and all subsequent cycles
**Secondary Prophylaxis – to prevent further neutropenia**: G-CSF prescribed in all subsequent chemotherapy cycles after the patient experienced neutropenia/low neutrophil count
**Reactive Treatment – to treat neutropenia**: G-CSF only prescribed in cycles where patient experiences neutropenia

*Please enter a number between 0 and 100 for each row.* Your responses must add to 100% in each column.
*Please consider your patients across both adjuvant and metastatic settings.*

|  |  | **% of Breast Cancer patients** | **% of Colorectal Cancer patients** | **% of Non-Small Cell Lung Cancer patients** | **% of Ovarian Cancer patients** | **% of patients with other solid tumors** | **% of Non-Hodgkin’s Lymphoma** | **% of patients with other hematologic malignancies** |
| --- | --- | --- | --- | --- | --- | --- | --- | --- |
| 1 | Received G-CSF as **Primary** Prophylaxis |  |  |  |  |  |  |  |
| 2 | Received G-CSF as **Secondary** Prophylaxis |  |  |  |  |  |  |  |
| 3 | Received G-CSF as **Reactive** Treatment |  |  |  |  |  |  |  |
| 4 | Received **No** G-CSF Therapy |  |  |  |  |  |  |  |
|  | TOTAL |  |  |  |  |  |  |  |

# Section B: Drivers of G-CSF Use

Next, we would like to understand your decision-making process regarding G-CSF prescribing overall and among different patient types.

**B1a.**

Which of the following clinical pathways / protocols does your office/institution primarily follow regarding G-CSF usage?

*Please select all that apply.*

| **1** | NCCN Guidelines |
| --- | --- |
| **2** | ASCO Guidelines |
| **3** | Institution/practice-level policy |
| **4** | ASH Guidelines |
| **5** | CDC Guidelines |
| **95** | Other (specify) |
| **96** | None |

B1.

To what extent does your **primary practice site** offer and encourage you to follow specific treatment pathways/protocols regarding how you use **G-CSF therapies**?

| 1 | **Strongly Encouraged** – I am strongly encouraged to follow specific clinical pathways / protocols regarding G-CSF use for most/all patients |
| --- | --- |
| 2 | **Somewhat Encouraged** – Pathways / protocols are provided at my practice for guidance, but I can deviate from them when I feel it is needed |
| 3 | **Not Encouraged At All** – Pathways / protocols are provided at my practice for guidance, but I am not required to follow them |

**B2.**

When assessing a patients **risk of developing febrile neutropenia** at the start of chemotherapy, how important is each of the following characteristics in determining whether a patient is **high risk (>20% risk of FN)**?

*You may either enter a number between 0-100 to reflect your rating or move the slider to a point on the continuum that best represents your rating.*

|  |  | Not at all important  0 |  | Extremely important  100 |
| --- | --- | --- | --- | --- |
| **1** | Type of cancer/disease | 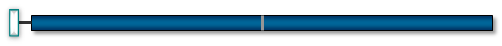 | | |
| **2** | Chemotherapy regimen | 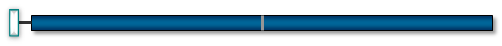 | | |
| **3** | Patient age | 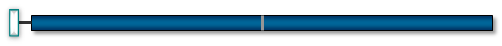 | | |
| **4** | Previous chemotherapy or radiation therapy | 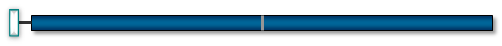 | | |
| **5** | Preexisting neutropenia or bone marrow involvement with tumor | 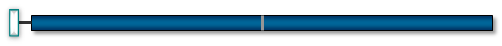 | | |
| **6** | Prior experience of neutropenia | 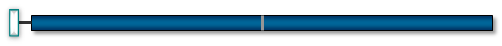 | | |
| **7** | Infection/open wounds | 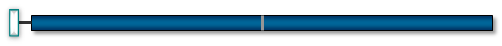 | | |
| **8** | Recent surgery | 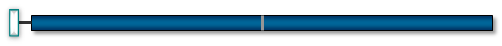 | | |
| **9** | Poor performance status | 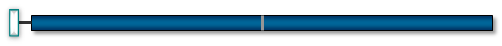 | | |
| **10** | Poor renal function | 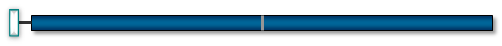 | | |
| **11** | Liver dysfunction | 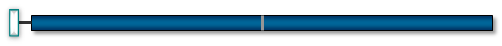 | | |
| **12** | Intent of chemotherapy | 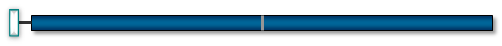 | | |
| **13** | Other (specify) | 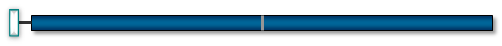 | | |

B3.

What, if anything, prevents you from prescribing any **G-CSF therapy at all as prophylaxis** at the **start of chemotherapy**, to a patient you believe to be at **high risk** of developing febrile neutropenia?
*Select all that apply.*

|  |  |
| --- | --- |
| **1** | Not on protocol/Not supported by guidelines |
| **2** | Cost to patient |
| **3** | Safety/side effect concerns |
| **4** | Concerns about insurance coverage |
| **5** | Lack of reimbursement to practice/organization |
| **6** | Patient inconvenience |
| **7** | Patient refusal |
| **8** | I prefer to wait and see |
| **95** | Other (Please Specify) |
| **99** | There are no barriers to prescribing G-CSF as prophylaxis to a high risk patient |

**B4a.**

To what extent does each of the following patient characteristics influence whether or not you prescribe a GCSF as primary prophylaxis at the start of chemotherapy?

Please allocate 100 points across each of the factors listed below. Please assign more points to the reasons that are of greater importance. You may use any number from 0 to 100.

|  |  | Allocate 100 Points |
| --- | --- | --- |
| 1 | High dose / Dose dense chemotherapy |  |
| 2 | Age 65+ |  |
| 3 | Prior experience of neutropenia |  |
| 4 | ECOG 2+ |  |
| 5 | Curative treatment |  |
|  | TOTAL |  |

B5.

Please rate your level of agreement with the following statements related to G-CSF treatments.

|  | Strongly disagree |  |  | |  |  | | |  | | Strongly agree |
| --- | --- | --- | --- | --- | --- | --- | --- | --- | --- | --- | --- |
|  | 1 | 2 | 3 | | 4 | 5 | | | 6 | | 7 |
| All patients at high risk of febrile neutropenia should receive a G-CSF as prophylaxis from the first cycle |  |  | |  | | |  |  | |  |  |
| I won’t prescribe a G-CSF until the patient shows signs of developing neutropenia |  |  | |  | | |  |  | |  |  |
| G-CSF therapy is underutilized among patients with a high risk of febrile neutropenia |  |  | |  | | |  |  | |  |  |
| G-CSF therapy is over utilized among patients with a low risk of febrile neutropenia |  |  | |  | | |  |  | |  |  |
| The benefits of G-CSF therapy outweigh the potential adverse effects |  |  | |  | | |  |  | |  |  |
| I am not able to prescribe G-CSF therapy as much as I would like due to protocol/guideline limitations |  |  | |  | | |  |  | |  |  |
| I am not able to prescribe G-CSF therapy as much as I would like due to payer/insurance restrictions |  |  | |  | | |  |  | |  |  |
| I prefer to monitor a patient’s neutrophil count in the first cycle before determining if a GCSF is necessary |  |  | |  | | |  |  | |  |  |
| It’s not always clear whether primary prophylaxis is indicated |  |  | |  | | |  |  | |  |  |

# Section C: Product Selection

Now we would like to understand more about your treatment selection of particular G-CSF therapies in your practice setting(s).

**C1a.**

Thinking back to the **[insert s11]** total patients you have seen or treated that have completed a course of chemotherapy within the past 6 months, how many were treated in each of the following primary practice settings?

|  |  | # of Patients |
| --- | --- | --- |
| 1 | Seen/Treated Primarily in Hospital |  |
| 2 | Seen/Treated Primarily in Office |  |
|  | TOTAL |  |

C1.

Thinking about all of the chemotherapy patients that you initiate on a G-CSF in your hospital, what proportion of them are first initiated on a G-CSF as an in-patient vs. an out-patient?

*Please enter a number between 0 and 100 for each row.* *Your responses must total 100%*

|  |  | % of Patients |
| --- | --- | --- |
| 1 | In-patient |  |
| 2 | Out-patient |  |
|  | TOTAL |  |

**C2.**

Which of the following brands of G-CSF have you **ever personally prescribed**?

*Select all that apply.*

|  |  | Ever Prescribed |
| --- | --- | --- |
| 1 | Neulasta® (pegfilgrastim) Pre-Filled Syringe (PFS) | 🞏 |
| 2 | Neulasta® (pegfilgrastim) Onpro® | 🞏 |
| 3 | Neupogen® (filgrastim) | 🞏 |
| 4 | Granix® (tbo-filgrastim) | 🞏 |
| 5 | Zarxio® (filgrastim-sndz) | 🞏 |
| 7 | Leukine (sargramostim) | 🞏 |

**C3.**

How much influence do **you personally** have on the decision in your **hospital/practice** about which specific G-CSF brands can be prescribed? Would you say you have…

|  |  | In Hospital Practice | In Office Practice |
| --- | --- | --- | --- |
| 1 | No influence at all | ○ | ○ |
| 2 | Some influence | ○ | ○ |
| 3 | A lot of influence | ○ | ○ |

C4.

Which of the following brands of G-CSF are available in your hospital for you to prescribe to **in-patients and/or out-patients**?

*Select all that apply for each column.*

|  |  | Available to Prescribe to In-Patients | Available to Prescribe to Out-Patients |
| --- | --- | --- | --- |
| 1 | Neulasta® (pegfilgrastim) PFS | 🞏 | 🞏 |
| 2 | Neulasta® (pegfilgrastim) Onpro® | 🞏 | 🞏 |
| 3 | Neupogen® (filgrastim) | 🞏 | 🞏 |
| 4 | Granix® (tbo-filgrastim) | 🞏 | 🞏 |
| 5 | Zarxio® (filgrastim-sndz) | 🞏 | 🞏 |
| 7 | Leukine (sargramostim) | 🞏 | 🞏 |
| 96 | None of the above | ○ | ○ |

**C4a.**

At your hospital, do you have any **restrictions** on your prescribing of **certain G-CSF brands** to **in-patients or to out-patients** (e.g. can only be prescribed in certain situations or to certain patient types)?

|  |  | Restrictions on Prescribing to In-Patients | Restrictions on Prescribing to Out-Patients |
| --- | --- | --- | --- |
| 1 | Neulasta® (pegfilgrastim) PFS | 🞏 | 🞏 |
| 2 | Neulasta® (pegfilgrastim) Onpro® | 🞏 | 🞏 |
| 3 | Neupogen® (filgrastim) | 🞏 | 🞏 |
| 4 | Granix® (tbo-filgrastim) | 🞏 | 🞏 |
| 5 | Zarxio® (filgrastim-sndz) | 🞏 | 🞏 |
| 7 | Leukine (sargramostim) | 🞏 | 🞏 |
| 96 | No restrictions | ○ | ○ |

**C4b.**

You indicated there are restrictions on your prescribing of the following brands in your hospital. What are the restrictions for each brand in each setting?

*Please describe in as much detail as possible.*

C5.

Which of the following brands of G-CSF are available for you to prescribe to **patients at your office practice**?

*Select all that apply.*

|  |  | Available to Prescribe |
| --- | --- | --- |
| 1 | Neulasta® (pegfilgrastim) PFS | 🞏 |
| 2 | Neulasta® (pegfilgrastim) Onpro® | 🞏 |
| 3 | Neupogen® (filgrastim) | 🞏 |
| 4 | Granix® (tbo-filgrastim) | 🞏 |
| 5 | Zarxio® (filgrastim-sndz) | 🞏 |
| 7 | Leukine (sargramostim) | 🞏 |
| 96 | None of the above | ○ |

**C5a.**

At your **office practice**, do you have **any restrictions** on your prescribing of **certain G-CSF brands** (e.g. can only be prescribed in certain situations or to certain patient types)?

|  |  | Restrictions on Prescribing |
| --- | --- | --- |
| 1 | Neulasta® (pegfilgrastim) PFS | 🞏 |
| 2 | Neulasta® (pegfilgrastim) Onpro® | 🞏 |
| 3 | Neupogen® (filgrastim) | 🞏 |
| 4 | Granix® (tbo-filgrastim) | 🞏 |
| 5 | Zarxio® (filgrastim-sndz) | 🞏 |
| 7 | Leukine (sargramostim) | 🞏 |
| 96 | None of the above | ○ |

**C5b.**

You indicated there are restrictions on your prescribing of the following brands in your office practice. What are the restrictions for each brand?

*Please describe in as much detail as possible.*

**C6.**

Thinking back to all the patients you have treated with chemotherapy in the past 6 months who received G-CSF therapy, what percentage received each of the following G-CSF brands in each setting?

*Please enter a number between 0 and 100 for each row.* *Your responses must add to 100% in each column.*

|  |  | % of Patients  Received each G-CSF in Hospital | % of Patients Received each G-CSF in Office |
| --- | --- | --- | --- |
| 1 | Neulasta® (pegfilgrastim) PFS |  |  |
| 2 | Neulasta® (pegfilgrastim) Onpro® |  |  |
| 3 | Neupogen® (filgrastim) |  |  |
| 4 | Granix® (tbo-filgrastim) |  |  |
| 5 | Zarxio® (filgrastim-sndz) |  |  |
| 7 | Leukine (sargramostim) |  |  |
| 95 | Other (specify): |  |  |
|  | TOTAL |  |  |

C7.

Of your patients treated with each G-CSF brand in your hospital practice, what proportion received each brand as primary prophylaxis, secondary prophylaxis, or reactive treatment?

*Click here to see definitions again*

*Please enter a number between 0 and 100 for each row.* *Your responses in each column must add to 100%*

|  |  | % of Neulasta® PFS Patients in Hospital |  | % of Neulasta® Onpro® Patients in Hospital |  | % of Neupogen® Patients in Hospital |  | % of Granix® Patients in Hospital |  | % of Zarxio® Patients in Hospital |  | % of Leukine Patients in Hospital | |
| --- | --- | --- | --- | --- | --- | --- | --- | --- | --- | --- | --- | --- | --- |
| 1 | Received as **Primary** Prophylaxis |  |  |  |  |  |  |  |  |  |  |  | |
| 2 | Received as **Secondary** Prophylaxis |  |  |  |  |  |  |  |  |  |  |  | |
| 3 | Received as **Reactive** Treatment |  |  |  |  |  |  |  |  |  |  | |  |
|  | TOTAL |  |  |  |  |  |  |  |  |  |  | |  |

C7a.

Of your patients treated with each G-CSF brand in your office practice, what proportion received each brand as primary prophylaxis, secondary prophylaxis, or reactive treatment?

*Click here to see definitions again*

*Please enter a number between 0 and 100 for each row.* *Your responses in each column must add to 100%*

|  |  | % of Neulasta® PFS Patients in Office |  | % of Neulasta® Onpro® Patients in Office |  | % of Neupogen® Patients in Office |  | % of Granix® Patients in Office |  | % of Zarxio® Patients in Office |  | % of Leukine Patients in Office | |
| --- | --- | --- | --- | --- | --- | --- | --- | --- | --- | --- | --- | --- | --- |
| 1 | Received as **Primary** Prophylaxis |  |  |  |  |  |  |  |  |  |  |  | |
| 2 | Received as **Secondary** Prophylaxis |  |  |  |  |  |  |  |  |  |  |  | |
| 3 | Received as **Reactive** Treatment |  |  |  |  |  |  |  |  |  |  | |  |
|  | TOTAL |  |  |  |  |  |  |  |  |  |  | |  |

**C8.**

Next we would like to understand your motivation for selecting a particular brand of G-CSF.

Please rate how important each of the following factors is in your decision to select a **specific G-CSF brand** to prescribe.

*You may either enter a number between 0-100 to reflect your rating or move the slider to a point on the continuum that best represents your rating.*

|  |  | Not at all important  0 |  | Extremely important  100 |
| --- | --- | --- | --- | --- |
| **1** | Out of pocket cost to patient | 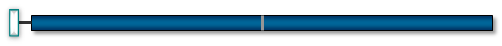 | | |
| **2** | Preferred agent on formulary | 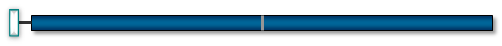 | | |
| **3** | On protocol/supported by guidelines | 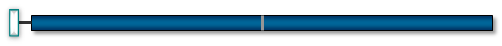 | | |
| **4** | Ease of payer approval | 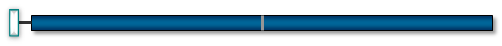 | | |
| **5** | Safety/side effect profile | 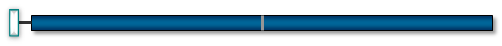 | | |
| **6** | Positive experience with the product | 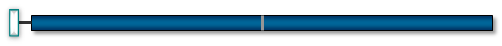 | | |
| **7** | Co-pay card/Patient support program | 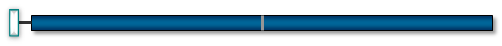 | | |
| **9** | Appropriate for patients at high risk of febrile neutropenia | 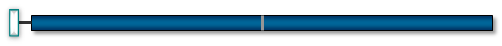 | | |
| **10** | Patient convenience | 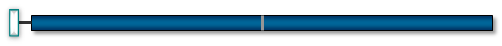 | | |
| **11** | Suitable for use as primary prophylaxis | 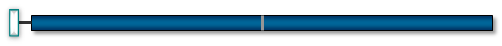 | | |
| **12** | Can be self-administered by the patient | 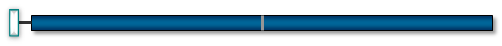 | | |
| **13** | Suitable for patients with active lifestyle | 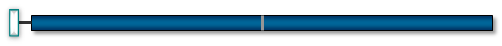 | | |

C9.

Please indicate your **level of satisfaction** with each of the following G-CSF therapies based on your own experience or what you have heard.

|  |  | Not at all satisfied |  |  |  |  |  | Extremely satisfied |
| --- | --- | --- | --- | --- | --- | --- | --- | --- |
|  |  | **1** | **2** | **3** | **4** | **5** | **6** | **7** |
| 1 | Neulasta® (pegfilgrastim) PFS |  |  |  |  |  |  |  |
| 2 | Neulasta® (pegfilgrastim) Onpro® |  |  |  |  |  |  |  |
| 3 | Neupogen® (filgrastim) |  |  |  |  |  |  |  |
| 4 | Granix® (tbo-filgrastim) |  |  |  |  |  |  |  |
| 5 | Zarxio® (filgrastim-sndz) |  |  |  |  |  |  |  |

C10.

Now we would like you to rate your perceptions of each product on each of the attributes you just reviewed. Based on what you know or have heard, **how does each G-CSF therapy perform** on each of the product attributes below?

|  |  | Performs very poorly |  |  | |  | |  | |  | | Performs very well |
| --- | --- | --- | --- | --- | --- | --- | --- | --- | --- | --- | --- | --- |
|  |  | **1** | **2** | **3** | | **4** | | **5** | | **6** | | **7** |
| 1 | Neulasta® (pegfilgrastim) PFS |  |  | |  | |  | |  | |  |  |
| 2 | Neulasta® (pegfilgrastim) Onpro® |  |  | |  | |  | |  | |  |  |
| 3 | Neupogen® (filgrastim) |  |  |  | |  | |  | |  | |  |
| 4 | Granix® (tbo-filgrastim) |  |  |  | |  | |  | |  | |  |
| 5 | Zarxio® (filgrastim-sndz) |  |  |  | |  | |  | |  | |  |

| 1 | Out-of-pocket cost to patient |
| --- | --- |
| 2 | Preferred agent on formulary |
| 3 | On protocol/supported by guidelines |
| 4 | Ease of payer approval |
| 5 | Safety/side effect profile |
| 6 | Positive experience with the product |
| 7 | Co-pay card/Patient support program |
| 9 | Appropriate for patients at high risk of febrile neutropenia |
| 10 | Patient convenience |
| 11 | Suitable for use as primary prophylaxis |
| 12 | Can be self-administered by the patient |
| 13 | Suitable for patients with active lifestyle |

**C11.**

Thinking about your overall use of G-CSF in your patients **over the last 1-2 years**, would you say that your overall prescribing of G-CSF has…

| Decreased significantly | Decreased somewhat | Remained the same | Increased somewhat | Increased significantly |
| --- | --- | --- | --- | --- |
| **1** | **2** | **3** | **4** | **5** |
|  |  |  |  |  |

**C11a.**

For what reason(s) has your G-CSF prescribing **decreased** over the last 1-2 years?
*Please describe in as much detail as possible.*

**C11b.**

For what reason(s) has your G-CSF prescribing **increased** over the last 1-2 years?
*Please describe in as much detail as possible.*

**C12.**

Thinking about your use of specific **G-CSF brands** in your patients, how has your usage of each G-CSF brand changed **over the last 1-2 years**?

*Please select one response for each column.*

|  |  | Decreased significantly | Decreased somewhat | Remained the same | Increased somewhat | Increased significantly |
| --- | --- | --- | --- | --- | --- | --- |
|  |  | **1** | **2** | **3** | **4** | **5** |
| **1** | Neulasta® (pegfilgrastim) PFS |  |  |  |  |  |
| **2** | Neulasta® (pegfilgrastim) Onpro® |  |  |  |  |  |
| **3** | Neupogen® (filgrastim) |  |  |  |  |  |
| **4** | Granix® (tbo-filgrastim) |  |  |  |  |  |
| **5** | Zarxio® (filgrastim-sndz) |  |  |  |  |  |

**C12a.**

For what reason(s) has your **prescribing of** [insert if C12_1=1-2: **Neulasta® PFS]** [insert if both C12_1=1-2 and C12_2=1-2: **and**] [insert if C12_2=1-2: **Neulasta® Onpro®] decreased** over the last 1-2 years?
*Please select all that apply for each column.*

|  |  | **Neulasta® (pegfilgrastim) PFS** **Decreased** | **Neulasta® (pegfilgrastim) Onpro® Decreased** |
| --- | --- | --- | --- |
| **1** | High out-of-pocket cost to patient | 🞏 | 🞏 |
| **2** | Change in formulary status (other brand preferred) | 🞏 | 🞏 |
| **3** | Not on protocol/supported by guidelines | 🞏 | 🞏 |
| **4** | Payer approval easier for other brands | 🞏 | 🞏 |
| **5** | Safety/side effect profile | 🞏 | 🞏 |
| **6** | Prefer/encouraged to use biosimilar G-CSF brands | 🞏 | 🞏 |
| **7** | Better co-pay support program offered by competitive G-CSF manufacturers | 🞏 | 🞏 |
| **8** | Contract with manufacturer or GPO offers better deal for competing agent | 🞏 | 🞏 |
| **9** | Reimbursement to practice/organization | 🞏 | 🞏 |
| **10** | Decrease in patient volume | 🞏 | 🞏 |
| **11** | Inconvenient for patients | 🞏 | 🞏 |
| **12** | Patients want an active lifestyle | 🞏 | 🞏 |
| **13** | Lack of caregiver support | 🞏 | 🞏 |
| **14** | Switching patients to Neulasta® Onpro® | 🞏 |  |
| **95** | Other (Please Specify) | 🞏 | 🞏 |

**C12b.**

For what reason(s) has your **prescribing of** [insert if C12_1=4-5: **Neulasta® PFS**] [insert if both C12_1=4-5 and C12_2=4-5: **and**] [insert if C12_2=4-5: **Neulasta® Onpro®**] **increased** over the last 1-2 years?
*Please select all that apply for each column.*

|  |  | **Neulasta® (pegfilgrastim) PFS** **Increased** | **Neulasta® (pegfilgrastim) Onpro® Increased** |
| --- | --- | --- | --- |
| **1** | Low out-of-pocket cost to patient | 🞏 | 🞏 |
| **2** | Change in formulary status (preferred agent) | 🞏 | 🞏 |
| **3** | On protocol/supported by guidelines | 🞏 | 🞏 |
| **4** | Covered by the majority of payers across the nation | 🞏 | 🞏 |
| **5** | Safety/side effect profile | 🞏 | 🞏 |
| **6** | Less experience/lower level of trust with biosimilars | 🞏 | 🞏 |
| **7** | Availability of co-pay support program to reduce patient out-of-pocket costs | 🞏 | 🞏 |
| **8** | Received attractive contract with manufacturer or GPO | 🞏 | 🞏 |
| **9** | Reimbursement to practice/organization | 🞏 | 🞏 |
| **10** | Increase in patient volume | 🞏 | 🞏 |
| **11** | Convenient dosing | 🞏 | 🞏 |
| **12** | Patients want an active lifestyle | 🞏 | 🞏 |
| **13** | Lack of caregiver support | 🞏 | 🞏 |
| **95** | Other (Please Specify) | 🞏 | 🞏 |

**C13a.**

Now please think about the types of patients to whom you typically prescribe Neulasta® Onpro®. What are the top 3 patient characteristics that drive you to prescribe Onpro®?

*Please rank your top 3 factors by dragging and dropping each factor to the right.*

| 1 | Adjuvant |  | Rank 1 |
| --- | --- | --- | --- |
| 2 | Low risk |  | Rank 2 |
| 3 | Active lifestyle |  | Rank 3 |
| 4 | Limited caregiver support |  |  |
| 5 | Lives far from treatment center |  |  |
| 6 | Accessible on insurance |  |  |
| 7 | No cost/coverage issues |  |  |
| 8 | 2 week chemotherapy regimen |  |  |
| 9 | No radiation |  |  |
| 10 | Other (specify) |  |  |

C14c.

Please think about all of your patients that receive **Neulasta® (pegfilgrastim)** as **primary prophylaxis** during their **1^st^ line of chemotherapy**.

If you compared the patients that receive the **PFS** to those that receive **Onpro®**, is there a difference in how long those patients typically **persist** on G-CSF therapy throughout their 1^st^ line chemotherapy?

| 1 | Patients on **Neulasta® PFS** **are more likely to** **stop** taking G-CSF therapy before they have completed chemotherapy than those on Neulasta® Onpro® |
| --- | --- |
| 2 | Patients on **Neulasta® Onpro®**  **are more likely to** **stop** taking G-CSF therapy before they have completed chemotherapy than those on Neulasta® PFS |
| 3 | There is no difference |

**C15.**

Thinking of your current **Neulasta® (pegfilgrastim) PFS** prescribing, what proportion of your patients receive Neulasta® at each of the following time frames?

*Please enter a number between 0 and 100 for each row.* *Your responses must add to 100%.*

|  |  | % of G-CSF Patients Receive Neulasta® PFS |
| --- | --- | --- |
| 1 | Within 24 hours of completion of chemotherapy administration |  |
| 2 | Between 24-48 hours after completion of chemotherapy administration |  |
| 3 | Two - four days after completion of chemotherapy administration |  |
| 4 | Five or more days after completion of chemotherapy administration |  |
|  | TOTAL |  |

**C16.**

What is the current recommended timing for administering **Neulasta® (pegfilgrastim) PFS**?

*Please select one.*

| 1 | Within 24 hours of completion of chemotherapy administration |
| --- | --- |
| 2 | Between 24-48 hours after completion of chemotherapy administration |
| 3 | Two - four days after completion of chemotherapy administration |
| 4 | Five or more days after completion of chemotherapy administration |

The next set of questions relates to current G-CSF brands and their biosimilars. The reference medicine on which the biosimilar is based is referred to as the original brand.

**C17.**

Thinking about G-CSF therapies in general, how much do you agree or disagree with the following statements?

| Strongly Disagree |  |  |  |  |  | Strongly Agree |
| --- | --- | --- | --- | --- | --- | --- |
| **1** | **2** | **3** | **4** | **5** | **6** | **7** |
|  |  |  |  |  |  |  |

Randomize

| 1 | I prefer the original G-CSF brand over G-CSF biosimilar brands |
| --- | --- |
| 2 | G-CSF biosimilars are just as effective as the original brand |
| 3 | G-CSF biosimilars are just as safe as the original brand |
| 4 | The availability of G-CSF biosimilars has increased my G-CSF prescribing overall |
| 5 | There is not enough long-term clinical data on the use of G-CSF biosimilars |
| 6 | I am comfortable prescribing G-CSF biosimilars |

**C18.**

How, if at all, have the availability of biosimilars Granix® (tbo-filgrastim) and Zarxio® (filgrastim-sndz) impacted your prescribing of **Neupogen® (filgrastim)**?

| 1 | Biosimilars have completely replaced my **Neupogen® (filgrastim)** prescribing |
| --- | --- |
| 2 | Biosimilars have slightly decreased my **Neupogen® (filgrastim)** prescribing, but I continue to utilize all |
| 3 | Biosimilars have significantly decreased my **Neupogen® (filgrastim)** prescribing, but I continue to utilize all |
| 4 | Biosimilars have had no impact on my **Neupogen® (filgrastim)** prescribing |

**C19.**

How, if at all, have the availability of biosimilars Granix® (tbo-filgrastim) and Zarxio® (filgrastim-sndz) impacted your **current** prescribing of **Neulasta® (pegfilgrastim)**?

| 1 | Biosimilars have completely replaced my **Neulasta® (pegfilgrastim)** prescribing |
| --- | --- |
| 2 | Biosimilars have slightly decreased my **Neulasta® (pegfilgrastim)** prescribing, but I continue to utilize all |
| 3 | Biosimilars have significantly decreased my **Neulasta® (pegfilgrastim)** prescribing, but I continue to utilize all |
| 4 | Biosimilars have had no impact on my **Neulasta® (pegfilgrastim)** prescribing |

**C20.**

How, if at all, **do you expect** biosimilars Granix® (tbo-filgrastim) and Zarxio® (filgrastim-sndz) to impact your future prescribing of **Neulasta® (pegfilgrastim)**?

| 1 | Biosimilars will completely replace my **Neulasta® (pegfilgrastim)** prescribing |
| --- | --- |
| 2 | Biosimilars will slightly decrease my **Neulasta® (pegfilgrastim)** prescribing, but I will continue to utilize all |
| 3 | Biosimilars will significantly decrease my **Neulasta® (pegfilgrastim)** prescribing, but I will continue to utilize all |
| 4 | Biosimilars will have no impact on my **Neulasta® (pegfilgrastim)** prescribing |

**C25.**

Please indicate how familiar you are with the Oncology Care Model of your institution:

| Not at all Familiar |  |  |  |  |  | Very Familiar |
| --- | --- | --- | --- | --- | --- | --- |
| **1** | **2** | **3** | **4** | **5** | **6** | **7** |
|  |  |  |  |  |  |  |

**C26.**

Please describe how, if at all, the Oncology Care Model has changed your use of G-CSF, including when and how you decide to prescribe G-CSF therapy.
*Please describe in as much detail as possible.*

**C27.**

How much, if at all, does type of payer (e.g., commercial insurance, Medicare) **impact your use of G-CSF in the following ways**?

|  |  | Not at all |  |  |  |  |  | A great deal |
| --- | --- | --- | --- | --- | --- | --- | --- | --- |
|  |  | **1** | **2** | **3** | **4** | **5** | **6** | **7** |
| **1** | Primary Prophylaxis |  |  |  |  |  |  |  |
| **2** | Secondary Prophylaxis |  |  |  |  |  |  |  |
| **3** | Reactive Treatment |  |  |  |  |  |  |  |

# Section D: Future G-CSF Prescribing

Lastly, we would like to understand how you anticipate your G-CSF prescribing to change in the future.

**D1.**

How, if at all, do you expect your prescribing of each of the following G-CSF brands to change over the next 3-5 years? Would you say that your **prescribing of each G-CSF brand** is likely to…

|  |  | Decrease significantly | Decrease somewhat | Remain the same | Increase somewhat | Increase significantly |
| --- | --- | --- | --- | --- | --- | --- |
|  |  | **1** | **2** | **3** | **4** | **5** |
| **1** | Neulasta® (pegfilgrastim) PFS |  |  |  |  |  |
| **2** | Neulasta® (pegfilgrastim) Onpro® |  |  |  |  |  |
| **3** | Neupogen® (filgrastim) |  |  |  |  |  |
| **4** | Granix® (tbo-filgrastim) |  |  |  |  |  |
| **5** | Zarxio® (filgrastim-sndz) |  |  |  |  |  |

**D1a.**

For what reason(s) do you expect your **Neulasta® (pegfilgrastim) PFS** prescribing to **decrease** over the next 3-5 years? *Please describe in as much detail as possible.*

**D1b.**

For what reason(s) do you expect your **Neulasta® (pegfilgrastim) PFS** prescribing to **increase** over the next 3-5 years?
*Please describe in as much detail as possible.*

**D1c.**

For what reason(s) do you expect your **Neulasta® (pegfilgrastim) Onpro®** prescribing to **decrease** over the next 3-5 years? *Please describe in as much detail as possible.*

**D1d.**

For what reason(s) do you expect your **Neulasta® (pegfilgrastim) Onpro®** prescribing to **increase** over the next 3-5 years?
*Please describe in as much detail as possible.*

D5.

Thinking about the **next 12 months**, what percentage of your chemotherapy patients prescribed a G-CSF do you expect to be treated with each of the following brands **in your hospital practice**?

*Your previous responses for patients who received G-CSF therapy in the past 6 months are shown.*

|  |  | % G-CSF Patients in Past 6 Months in Hospital | % G-CSF Patients in Next 12 Months in Hospital |
| --- | --- | --- | --- |
| 1 | Neulasta® (pegfilgrastim) PFS |  |  |
| 2 | Neulasta® (pegfilgrastim) Onpro® |  |  |
| 3 | Neupogen® (filgrastim) |  |  |
| 4 | Granix® (tbo-filgrastim) |  |  |
| 5 | Zarxio® (filgrastim-sndz) |  |  |
| 7 | Leukine (sargramostim) |  |  |
| 95 | Other (specify): |  |  |
|  | TOTAL |  |  |

D5a.

Thinking about the **next 12 months**, what percentage of your chemotherapy patients prescribed a G-CSF do you expect to be treated with each of the following brands **in your office practice**?

*Your previous responses for patients who received G-CSF therapy in the past 6 months are shown.*

|  |  | % G-CSF Patients in Past 6 Months in Office | % G-CSF Patients in Next 12 Months in Office |
| --- | --- | --- | --- |
| 1 | Neulasta® (pegfilgrastim) PFS |  |  |
| 2 | Neulasta® (pegfilgrastim) Onpro® |  |  |
| 3 | Neupogen® (filgrastim) |  |  |
| 4 | Granix® (tbo-filgrastim) |  |  |
| 5 | Zarxio® (filgrastim-sndz) |  |  |
| 7 | Leukine (sargramostim) |  |  |
| 95 | Other (specify): |  |  |
|  | TOTAL |  |  |

**C21.**

Please indicate your level of familiarity with any **pegfilgrastim** **biosimilars** in development:

| 1 | Not at all familiar |
| --- | --- |
| 2 | Somewhat familiar |
| 3 | Very familiar |

**C21a.**

Please list the name(s) of any companies you are aware of that are developing a **pegfilgrastim biosimilar**:

C22.

Now please assume pegfilgrastim is available as a biosimilar and approved for use. With that in mind, how much do you agree or disagree with the following statements?

| Strongly Disagree |  |  |  |  |  | Strongly Agree |
| --- | --- | --- | --- | --- | --- | --- |
| **1** | **2** | **3** | **4** | **5** | **6** | **7** |
|  |  |  |  |  |  |  |

| 1 | I would prefer the original Neulasta® (pegfilgrastim) over its biosimilar |
| --- | --- |
| 2 | Pegfilgrastim biosimilar would be just as effective as the original brand |
| 3 | Pegfilgrastim biosimilar would be just as safe as the original brand |
| 4 | The availability of a pegfilgrastim biosimilar would increase my G-CSF prescribing overall |

C24.

How much influence would **you personally** have on the decision in your **hospital/practice** about whether a **pegfilgrastim biosimilar** could be prescribed? Would you say you have…

|  |  | In Hospital Practice | In Office Practice |
| --- | --- | --- | --- |
| 1 | No influence at all | ○ | ○ |
| 2 | Some influence | ○ | ○ |
| 3 | A lot of influence | ○ | ○ |

**D3.**

**Please continue to assume pegfilgrastim is available as a biosimilar and approved for use.**

How, if at all, do you expect your **Neulasta® (pegfilgrastim)** **prescribing** to change over the next 3-5 years for the following **patient types based on risk level**? Would you say that your **prescribing of Neulasta®** is likely to…

|  |  | Decrease significantly | Decrease somewhat | Remain the same | Increase somewhat | Increase significantly |
| --- | --- | --- | --- | --- | --- | --- |
|  |  | **1** | **2** | **3** | **4** | **5** |
| **1** | High Risk (>20% risk for FN) |  |  |  |  |  |
| **2** | Intermediate Risk (10-20% risk for FN) |  |  |  |  |  |
| **3** | Low Risk (<10% risk for FN) |  |  |  |  |  |

**D4.**

**Please continue to assume pegfilgrastim is available as a biosimilar and approved for use.**

How, if at all, do you expect your **Neulasta® (pegfilgrastim)** **prescribing** to change over the next 3-5 years for the following **usage scenarios**? Would you say that your **prescribing of Neulasta®** as each type of use is likely to…

|  |  | Decrease significantly | Decrease somewhat | Remain the same | Increase somewhat | Increase significantly |
| --- | --- | --- | --- | --- | --- | --- |
|  |  | **1** | **2** | **3** | **4** | **5** |
| **1** | Primary Prophylaxis |  |  |  |  |  |
| **2** | Secondary Prophylaxis |  |  |  |  |  |
| **3** | Reactionary/ Reactive |  |  |  |  |  |

**D6.**

Please continue to assume **pegfilgrastim is available as a biosimilar** and approved for use.

With this in mind, again thinking about the **next 12 months**, what percentage of your chemotherapy patients prescribed a G-CSF do you expect to be treated with each of the following brands **in your hospital practice**?

*Your previous responses for patients who would receive G-CSF therapy in the next 12 months are shown.*

***Please assume the restrictions and access for this biosimilar are similar to current G-CSF biosimilars on the market.***

|  |  | % G-CSF Patients in Next 12 Months in Hospital | % G-CSF Patients Next 12 Months in Hospital with Pegfilgrastim Biosimilar Available |
| --- | --- | --- | --- |
| 6 | Pegfilgrastim biosimilar |  |  |
| 1 | Neulasta® (pegfilgrastim) PFS |  |  |
| 2 | Neulasta® (pegfilgrastim) Onpro® |  |  |
| 3 | Neupogen® (filgrastim) |  |  |
| 4 | Granix® (tbo-filgrastim) |  |  |
| 5 | Zarxio® (filgrastim-sndz) |  |  |
| 7 | Leukine (sargramostim) |  |  |
| 95 | Other (specify): |  |  |
|  | TOTAL |  |  |

**D6a.**

Please continue to assume **pegfilgrastim is available as a biosimilar** and approved for use.

With this in mind, again thinking about the **next 12 months**, what percentage of your chemotherapy patients prescribed a G-CSF do you expect to be treated with each of the following brands **in your office practice**?

*Your previous responses for patients who would receive G-CSF therapy in the next 12 months are shown.*

***Please assume the restrictions and access for this biosimilar are similar to current G-CSF biosimilars on the market.***

|  |  | % G-CSF Patients in Next 12 Months in Office | % G-CSF Patients Next 12 Months in Office with Pegfilgrastim Biosimilar Available |
| --- | --- | --- | --- |
| 6 | Pegfilgrastim biosimilar |  |  |
| 1 | Neulasta® (pegfilgrastim) PFS |  |  |
| 2 | Neulasta® (pegfilgrastim) Onpro® |  |  |
| 3 | Neupogen® (filgrastim) |  |  |
| 4 | Granix® (tbo-filgrastim) |  |  |
| 5 | Zarxio® (filgrastim-sndz) |  |  |
| 7 | Leukine (sargramostim) |  |  |
| 95 | Other (specify): |  |  |
|  | TOTAL |  |  |

# Section E: Demographics

Doctor, these last few questions are for classification purposes only.

E1.

Is your primary practice in an urban, suburban or rural area?

*Please select one response.*

| **1** | Urban |  |
| --- | --- | --- |
| **2** | Suburban |  |
| **3** | Rural |  |

E2.

Does your hospital/office practice use Electronic Medical Records (EMR)?

|  |  | EMR Used in Hospital | EMR Used in Office |
| --- | --- | --- | --- |
| 1 | Yes | ○ | ○ |
| 2 | No | ○ | ○ |

E3.

Please describe how, if at all, usage of EMR in your **hospital** impacts your prescribing of G-CSF brands.

E3b.

Please describe how, if at all, usage of EMR in your **office practice** impacts your prescribing of G-CSF brands.

E4.

Approximately what percent of your patients have each of the following types of health insurance?

|  |  | % of Patients |
| --- | --- | --- |
| 1 | Private insurance (such as an HMO or PPO) |  |
| 2 | Medicare |  |
| 3 | Medicaid |  |
| 4 | Veterans Affairs |  |
| 5 | No insurance coverage |  |
| 95 | Other |  |
| 96 | Unknown |  |
|  | TOTAL |  |

# CLOSING

The researchers who designed this study would like to hear your feedback about this survey, including how easy or difficult it was to take.

*Type your response below. Please be as specific as possible.*
